# Supplementary material for: A neonatal mouse model characterizes transmissibility of SARS-CoV-2 variants and reveals a role for ORF8
Source: Nat Commun. 2023 May 25;14:3026. doi: 10.1038/s41467-023-38783-0 (PMC10211296; doi:10.1038/s41467-023-38783-0)
Supplement: Supplementary file 1 — Supplementary Information [file 41467_2023_38783_MOESM1_ESM.pdf]

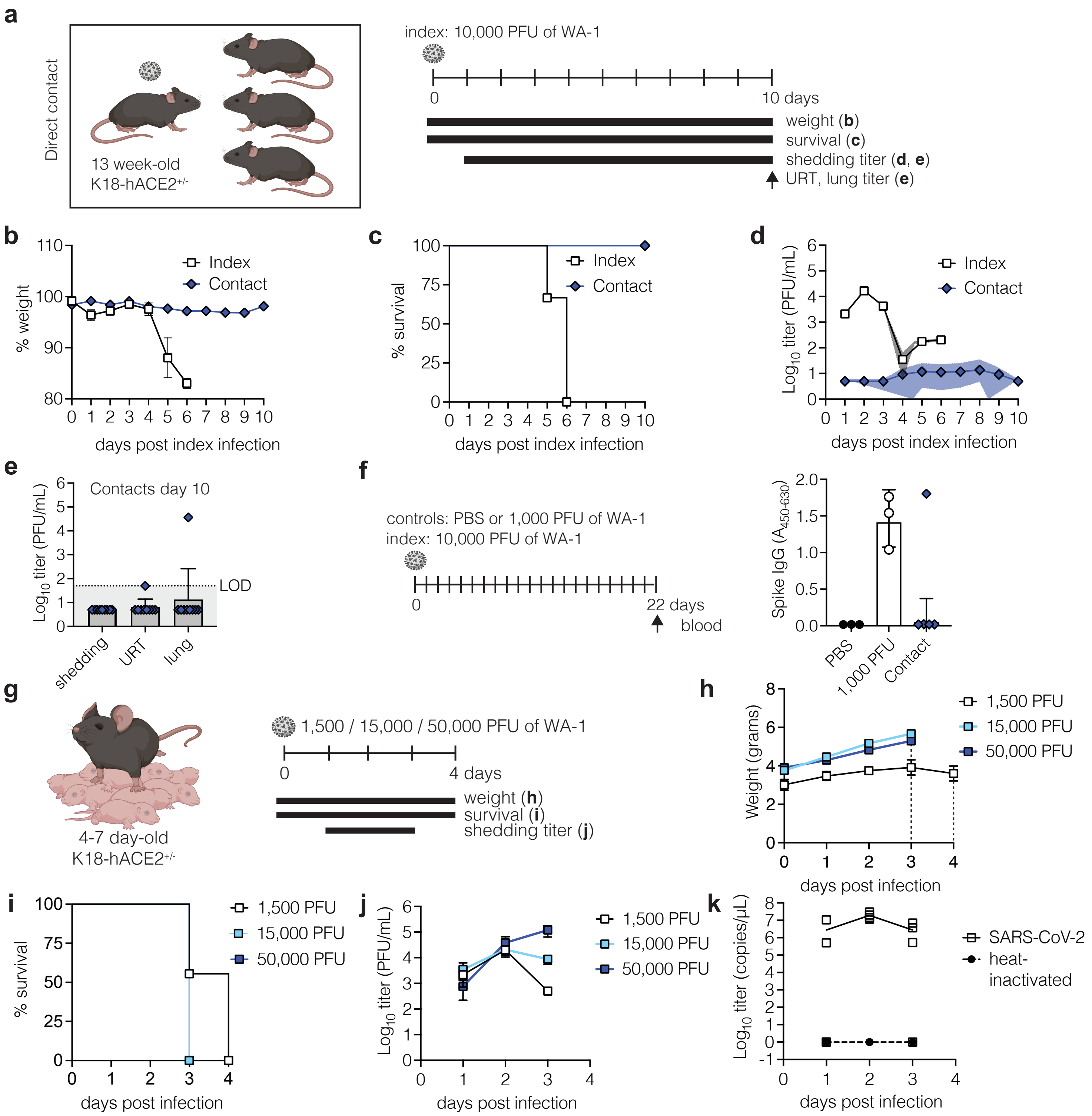

Supplemental Figure 1, related to Figure 1. a, Experimental overview for panels b-e. 13-week-old K18-hACE2$^{+/-}$ mice were infected intranasally with a lethal dose (10,000 PFU) of SARS-CoV-2 WA-1 (index) and cohoused with uninfected littermates (contact) for 10 days at an index:contact ratio of 1:3. Weight and survival were monitored daily. Viral shedding samples were collected by dipping the nares of each individual mice in viral medium daily. Endpoint titers from retrotracheal lavages and lung homogenates were taken at day 10. Data from n=3 cages each with n=1 index and n=3 contacts. **b**. Mean ± SEM body weight of index and contact mice. Created with BioRender.com. **c**. Survival of index and contact mice. **d.** Viral burden in shedding samples analyzed plaque assay for infectious virus. Data shown as geometric mean (line) with geometric standard deviation (shaded areas). Individual values below the limit of detection (LOD, 50 PFU/ml) were set to 5. **e.** Viral burden in contact shedding samples, retrotracheal lavage, or lungs at day 10. **f.** 13-week-old K18-hACE2$^{+/-}$ mice were infected intranasally with a lethal dose (10,000 PFU) of SARS-CoV-2 WA-1 (index) and cohoused with uninfected littermates (contact) for 22 days at an index:contact ratio of 1:2 or 1:3. Additional negative control animals were inoculated with PBS, and additional positive control animals with a sub-lethal dose of 1,000 PFU. Seroconversion was determined by ELISA for Spike IgG. Data shown as mean ± SEM for n=3 PBS-treated animals, n=3 1000 PFU positive controls, and n=5 contact animals. **g.** Experimental overview for panels h-j. 4 to 7 days old K18-hACE2$^{+/-}$ pups were infected intranasally with either 1,500, 15,000 or 50,000 PFU of SARS-CoV-2 WA-1 for 4 days. Weight and survival were monitored daily. Viral shedding samples were collected by dipping the nares of each individual pup in viral medium daily. Data from at least n=3 mice per group. Created with BioRender.com. **h**. Mean ± SEM body weight. Dotted lines represent last day of survival of each group. **i**. Survival **j.** Viral burden in shedding samples analyzed by plaque assay for infectious virus. Data shown as as mean ± SEM. **k.** 4 to 7 days old K18-hACE2$^{+/-}$ pups were infected intranasally with either 15,000 PFU of SARS-CoV-2 WA-1 or heat-inactivated SARS-CoV-2 WA-1 for 4 days. Viral burden in daily shedding samples was evaluated by RT-qPCR. Data shown as individual values (symbols) and mean (lines) from at least n=4 individual mice per group. Source data are provided in the Source Data file.

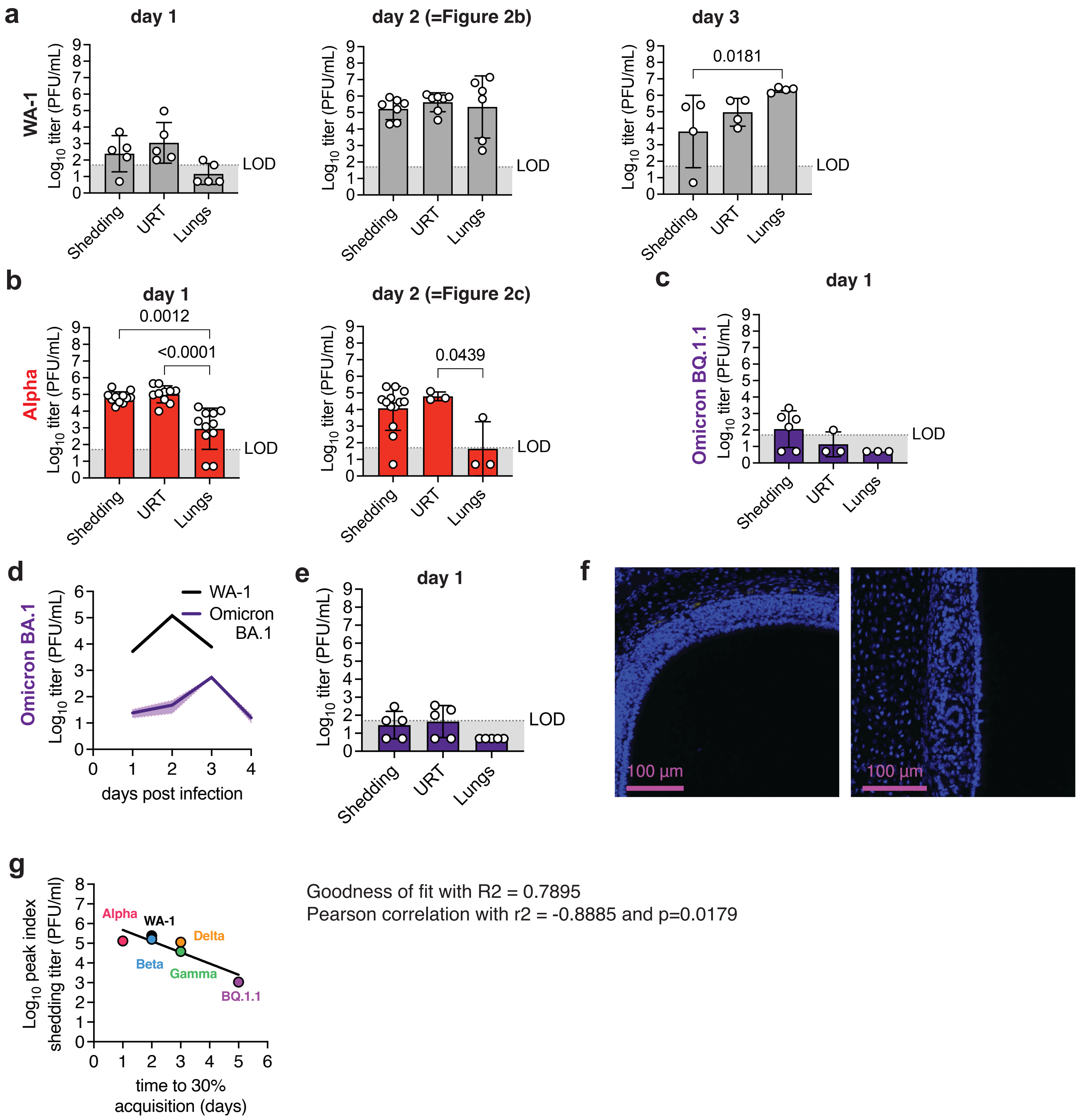

Supplemental Figure 2, related to Figures 2 and 3: SARS-CoV-2 replication dynamics and tropism in index mice. a.-c. 4-7 day old K18-hACE2$^{+/-}$ pups were infected intranasally with 1500 PFU of SARS-CoV-2 WA-1 (**a**), Alpha (**b**), or Omicron BQ.1.1 (**c**). At indicated days, we collected shedding titers by dipping the nares into collection medium and sacrificed some pups to collect retrotracheal lavages and lungs to determine viral infectious virus titers. Data of Shedding, URT and Lung titers are shown as symbols for individual pups; bars and error bars represent the geometric mean and geometric standard deviation, respectively. Data from at least n=3 pups per group. Individual values below the limit of detection (LOD, 50 PFU/ml) were set to 5. p-values were determined by Kruskal-Wallis test. Only significant values are presented. Day 2 panels are identical with those in Figure 2 a and c, respectively, and shown again here for easea of sice-by-side comparison. **d.-f.** 4-7 day old K18-hACE2$^{+/-}$ pups were infected intranasally with 1500 PFU of SARS-CoV-2 Omicron BA.1. **d.** Viral shedding samples were collected daily by dipping the nares of each individual pup in viral medium. Daily shedding data is shown as geometric mean (line) with geometric standard deviation (dotted lines). Day 0, inoculum. Data from at least 2 independent repetitions with n = 6 - 15 pups per group. **e.** At 1 dpi, we sacrificed some pups to collect retrotracheal lavages and lungs to determine viral infectious virus titers. Data of Shedding, URT and Lung titers are shown as symbols for individual pups; bars and error bars represent the geometric mean and geometric standard deviation, respectively. Data from at least n=5 pups per group. Individual values below the limit of detection (LOD, 50 PFU/ml) were set to 5. p-values were determined by Kruskal-Wallis test. Only significant values are presented. **f.** Immunohistochemistry for SARS-CoV-2 N at 1 dpi with Omicron BA.1 in neonatal mice nasopharynx. Heads were paraffin embedded, sectioned through the nasopharynx, and stained for SARS-CoV-2 N protein (yellow) and DAPI (blue) for nuclei. **g.** Peak index shedding titers (Fig 2) shown over time to 30 % acquisition in contacts (Fig 3). Best fit linear regression curves were fitted on data and goodness of fit (R2), Pearson correlation (r2) and two-tailed significance (p value) was calculated using GraphPad Prism 9.5 software. Souce data are provided as a Source Data file.

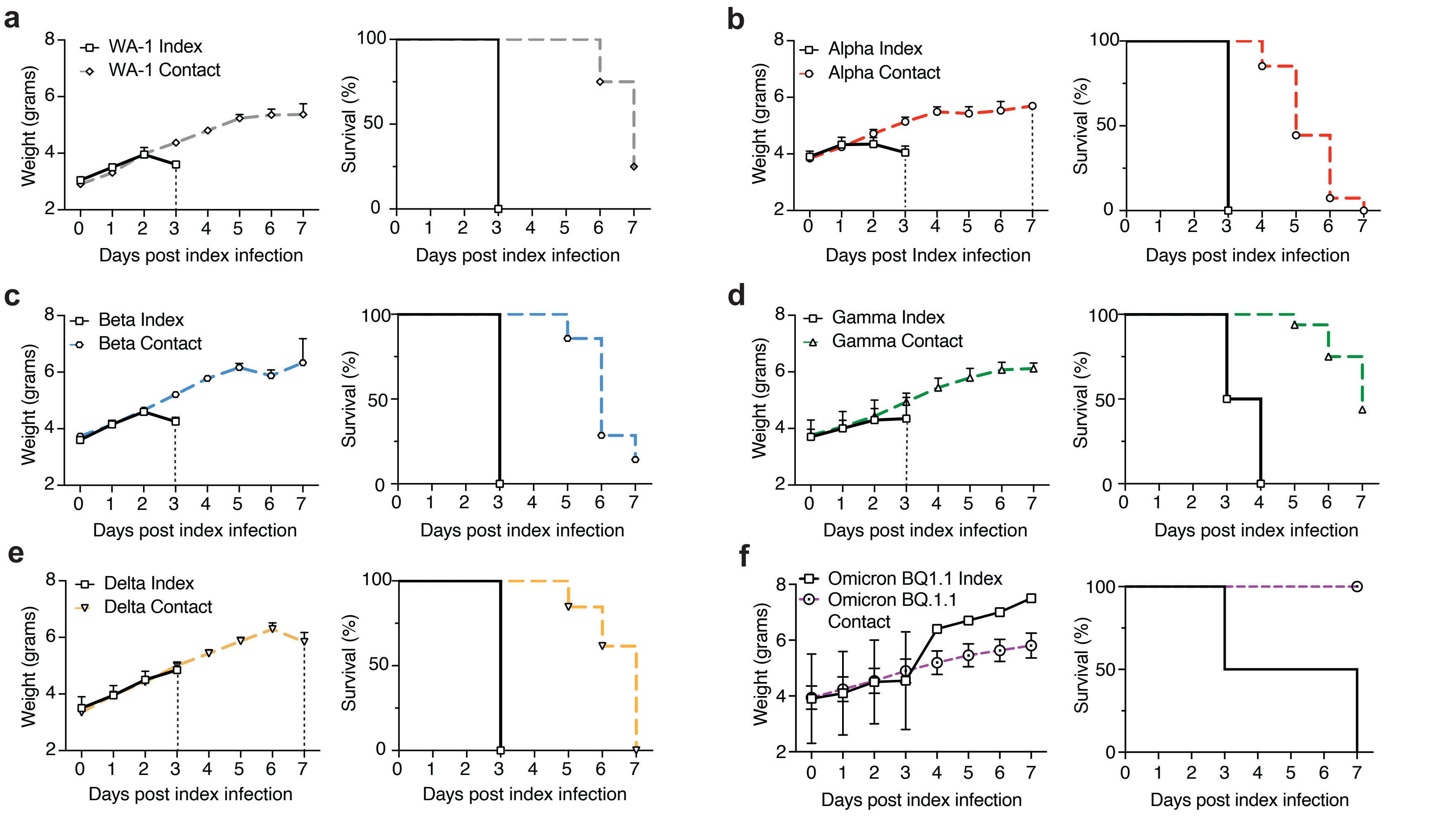

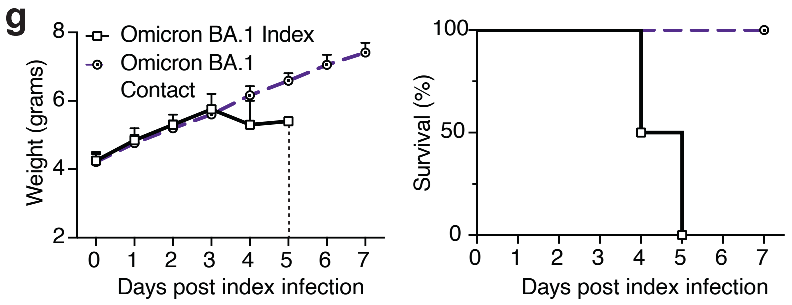

Supplemental Figure 3, related to Figure 3: Weight and survival curves of index and contact mice during transmission experiments with SARS-CoV-2 variants. a-g. 4 to 7 day-old index pups were infected intranasally with 1500 PFU of SARS-CoV-2 and cohoused with uninfected littermates (contact) for 7 days. Left panels: Survival of index and contact mice. Vertical dotted lines represent last day of survival of each group. Right panels: body weight curve of index and contact mice, represented as mean with error bars indicating SEM. index mice n = 2-4, contact mice n = 8 - 27. (**a**) WA-1, (**b**) Alpha, (**c**), Beta, (**d**) Gamma, (**e**) Delta, (**f**) Omicron BQ.1.1 or (**g**) Omicron BA.1. Source data are provided in the Source Data file.

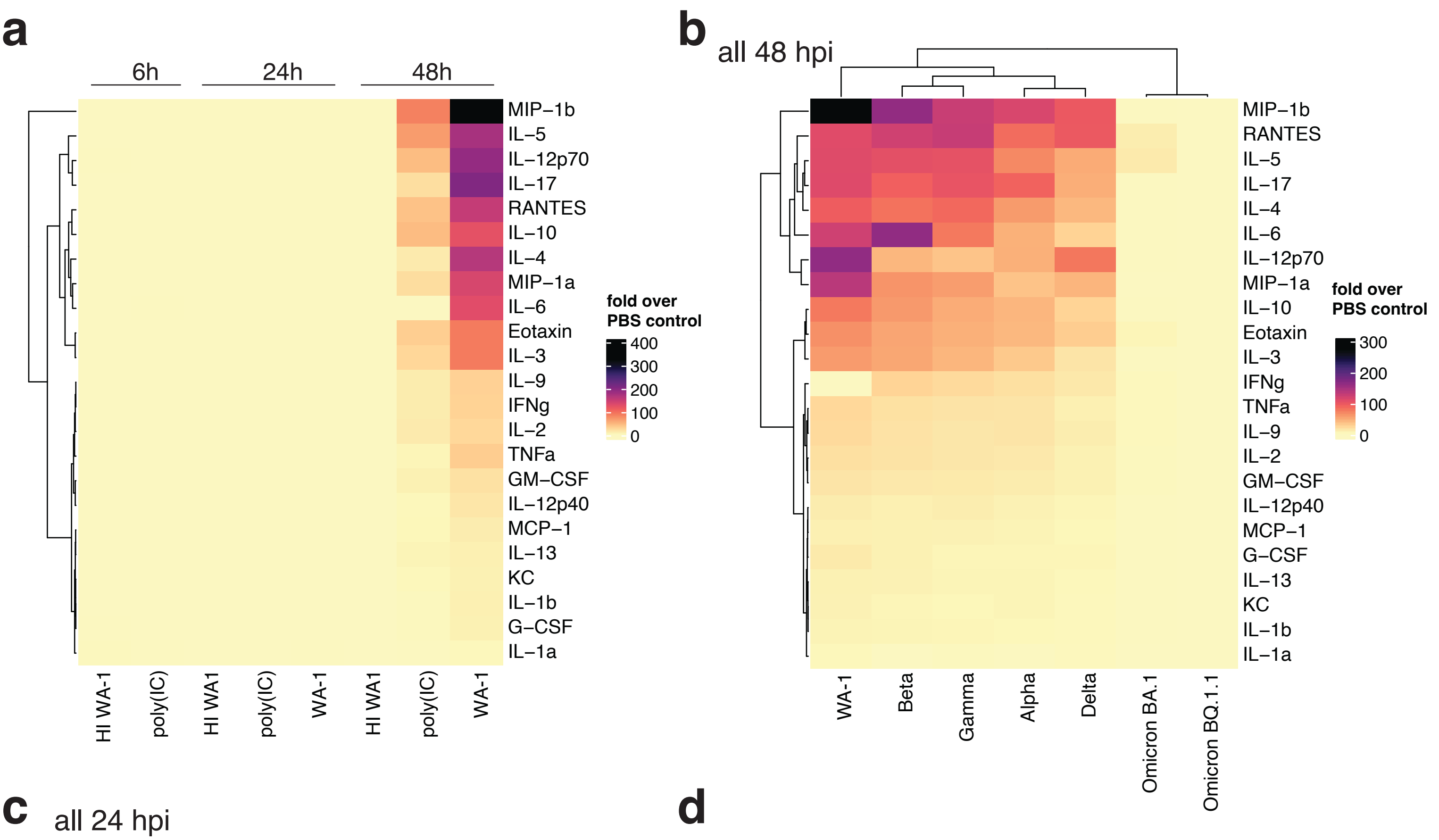

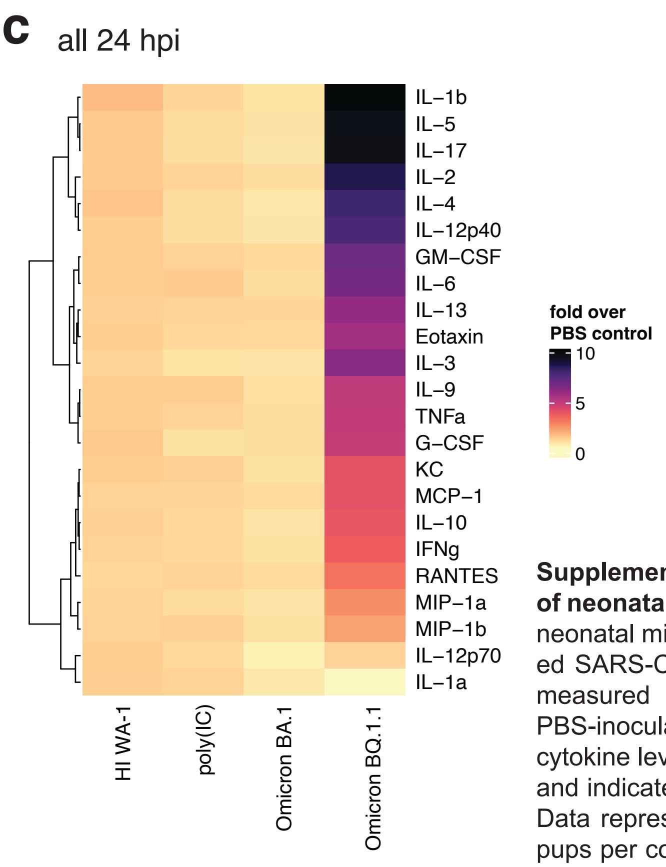

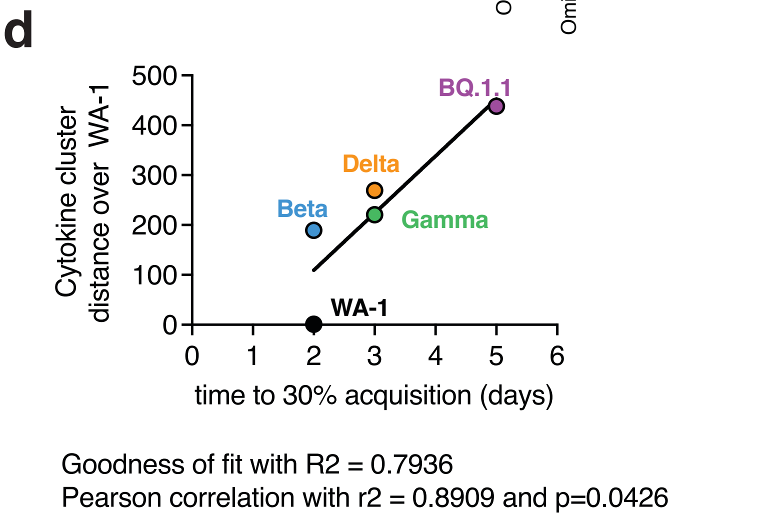

Supplemental Figure 4, related to Figure 3: Cytokine levels in shedding samples of neonatal mice. a, Heatmap representing cytokine levels in shedding samples from neonatal mice treated either intranasally with poly (I:C) or infected with heat inactivated SARS-CoV-2 WA-1 (HI) or infectious WA-1 at 6, 24, or 48 hours post-infection, measured by multiplex ELISA. Data represent -fold induction over values from PBS-inoculated pups. At least n=2 pups per condition. **b**, Heatmap representing cytokine levels in shedding samples of neonatal mice infected intranasally with WA-1 and indicated variants, at 48 hours post-infection and measured by multiplex ELISA. Data represent -fold induction over values from PBS-inoculated pups. At least n=2 pups per condition. **c.** Heatmap representing cytokine levels in shedding samples of neonatal mice infected with heat inactivated SARS-CoV-2 WA-1 (HI) or infectious SARS-CoV-2 Omicron BA.1 or BQ.1.1 variants, at 24 hours post-infection and measured by multiplex ELISA. Data represent -fold induction over values from PBS-inoculated pups. At least n=2 pups per condition. **d.** Cytokine cluster distance (panel b) shown over time to 30% acquisition in contacts (Fig. 3). Best fit linear regression curves were fitted on data and goodness of fit (R2), Pearson correlation (r2) and two-tailed significance (p value) was calculated using GraphPad Prism 9.5 software. Alpha was omitted from the analysis. Source data are provided in the Source Data file.

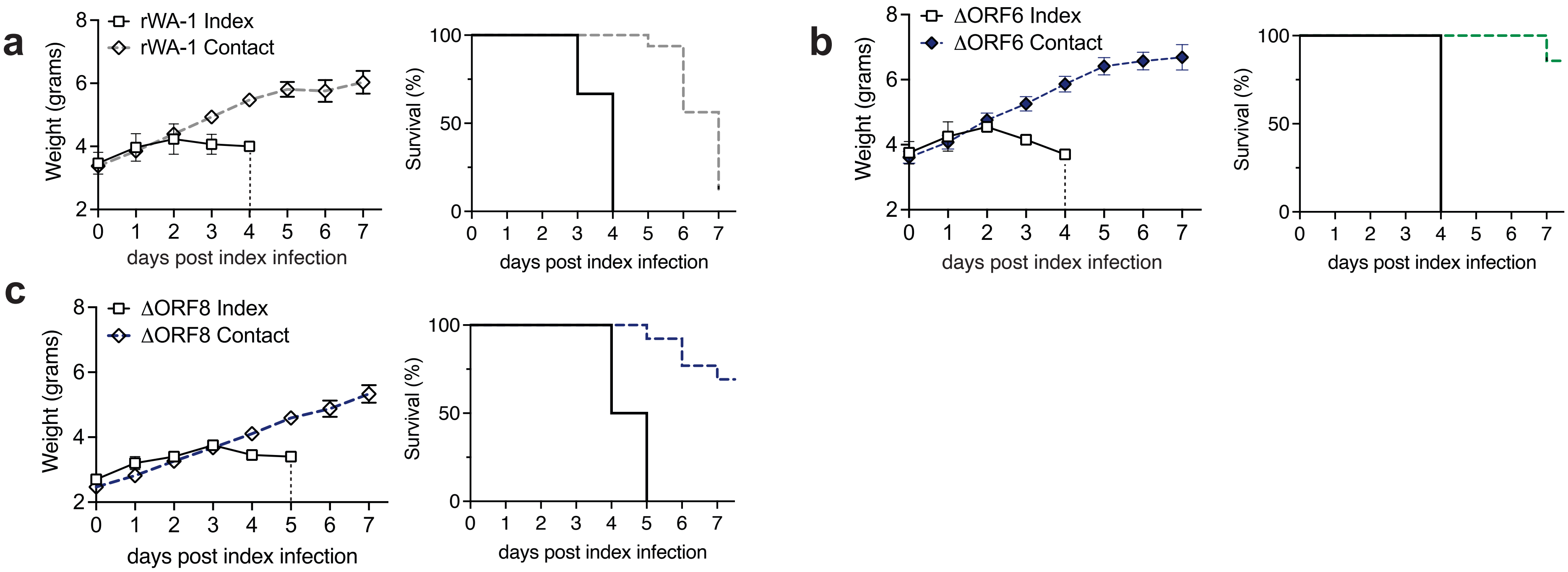

Supplemental Figure 5, related to Figure 4: Morbidity, mortality, and sites of upper respiratory viral replication for ΔORF6 and ΔORF8-infected neonatal mice. a-c, 4-7 day-old index pups were infected intranasally with 1500 PFU of indicated recombinant SARS-CoV-2 and cohoused with uninfected littermates (contact) for 7 days. Data corresponding to Figure 4. Left panels: Survival of index and contact mice. Vertical dotted lines represent the last day of survival of each group. Right panels: body weight curves of index and contact mice, represented as mean with error bars indicating SEM. Index mice n=2-4, contact mice n=8-15. (a) recombinant WA-1 (rWA-1), (b) ΔORF6, (c) ΔORF8. Source data are provided in the Source Data file.
