# Supplementary material for: A neonatal mouse model characterizes transmissibility of SARS-CoV-2 variants and reveals a role for ORF8
Source: Nat Commun. 2023 May 25;14:3026. doi: 10.1038/s41467-023-38783-0 (PMC10211296; doi:10.1038/s41467-023-38783-0)
Supplement: Supplementary file 3 — Reporting Summary [file 41467_2023_38783_MOESM3_ESM.pdf]

nature portfolio

Corresponding author(s): Dittmann, Ortigoza

Last updated by author(s): 05/05/2023

Reporting Summary

Nature Portfolio wishes to improve the reproducibility of the work that we publish. This form provides structure for consistency and transparency in reporting. For further information on Nature Portfolio policies, see our Editorial Policies and the Editorial Policy Checklist.

Statistics

For all statistical analyses, confirm that the following items are present in the figure legend, table legend, main text, or Methods section.

n/a	Confirmed	
☐	☒	The exact sample size (*n*) for each experimental group/condition, given as a discrete number and unit of measurement
☐	☒	A statement on whether measurements were taken from distinct samples or whether the same sample was measured repeatedly
☐	☒	The statistical test(s) used AND whether they are one- or two-sided<br>*Only common tests should be described solely by name; describe more complex techniques in the Methods section.*
☐	☒	A description of all covariates tested
☐	☒	A description of any assumptions or corrections, such as tests of normality and adjustment for multiple comparisons
☐	☒	A full description of the statistical parameters including central tendency (e.g. means) or other basic estimates (e.g. regression coefficient) AND variation (e.g. standard deviation) or associated estimates of uncertainty (e.g. confidence intervals)
☐	☒	For null hypothesis testing, the test statistic (e.g. *F*, *t*, *r*) with confidence intervals, effect sizes, degrees of freedom and *P* value noted<br>*Give P values as exact values whenever suitable.*
☒	☐	For Bayesian analysis, information on the choice of priors and Markov chain Monte Carlo settings
☐	☒	For hierarchical and complex designs, identification of the appropriate level for tests and full reporting of outcomes
☐	☒	Estimates of effect sizes (e.g. Cohen's *d*, Pearson's *r*), indicating how they were calculated

Our web collection on statistics for biologists contains articles on many of the points above.

Software and code

Policy information about availability of computer code

Data collection	QuantStudio software v1.4. was used for qPCR data collection.<br>xPONENT version 4.3.229.0 software was used for the cytokine data collection.<br>Research image data was managed using OMERO Plus v5.6 (Glencoe Software) for viewing, annotation and/or Figure making with OMERO.figure v 4.4 (OME team).
Data analysis	TThis study used commercially available Graphpad prism software v9.5.1(528) for data representation and statistical analysis. Cytokine data were analyzed and graphically displayed in clustered heatmaps, generated using the ComplexHeatmap package v.2.14.0 in program R. xPONENT version 4.3.229.0 software and Microsoft Excel v16.70 were used for the cytokine analysis. QuantStudio software v1.4. and Excel v16.70 were used for qPCR data analysis. Image analysis was done using InForm® version 2.6 software from Akoya Biosciences.

For manuscripts utilizing custom algorithms or software that are central to the research but not yet described in published literature, software must be made available to editors and reviewers. We strongly encourage code deposition in a community repository (e.g. GitHub). See the Nature Portfolio guidelines for submitting code & software for further information.

Data

Policy information about availability of data

All manuscripts must include a data availability statement. This statement should provide the following information, where applicable:

- Accession codes, unique identifiers, or web links for publicly available datasets
- A description of any restrictions on data availability
- For clinical datasets or third party data, please ensure that the statement adheres to our policy

All raw data of this study has been compiled in a Source Data file. Availability of the data in this Source Data file has been pointed out in the data availability statement.

Human research participants

Policy information about studies involving human research participants and Sex and Gender in Research.

Reporting on sex and gender	n/a
Population characteristics	n/a
Recruitment	n/a
Ethics oversight	n/a

Note that full information on the approval of the study protocol must also be provided in the manuscript.

Field-specific reporting

Please select the one below that is the best fit for your research. If you are not sure, read the appropriate sections before making your selection.

☒ Life sciences ☐ Behavioural & social sciences ☐ Ecological, evolutionary & environmental sciences

For a reference copy of the document with all sections, see nature.com/documents/nr-reporting-summary-flat.pdf

Life sciences study design

All studies must disclose on these points even when the disclosure is negative.

Sample size	No sample size calculations were performed to power each study. Sample sizes for mouse models were determined based on previous results from similar in vivo experiments which showed that the use of 5-12 mice per group represents a minimally sufficient sample to produce a study power of >80% (PMID: 30563897 and 32553273)
Data exclusions	No data was excluded in this study
Replication	All experiments were performed in at least 2 independent biological replicates. All attempts at replication were successful.
Randomization	Mice were randomly assigned to each treatment group (infection with different variants)
Blinding	Investigators were not blinded for the following analyses (morbidity, mortality and collection of shedding samples) due to the biosafety concerns associated with handling these samples. For determination of cytokine levels and for the determination of viral burden in shedding, lavages and organs, samples were blinded prior to analysis.

Reporting for specific materials, systems and methods

We require information from authors about some types of materials, experimental systems and methods used in many studies. Here, indicate whether each material, system or method listed is relevant to your study. If you are not sure if a list item applies to your research, read the appropriate section before selecting a response.

Materials & experimental systems

n/a	Involved in the study
☐	☒ Antibodies
☐	☒ Eukaryotic cell lines
☒	☐ Palaeontology and archaeology
☐	☒ Animals and other organisms
☒	☐ Clinical data
☒	☐ Dual use research of concern

Methods

n/a	Involved in the study
☒	☐ ChIP-seq
☒	☐ Flow cytometry
☒	☐ MRI-based neuroimaging

Antibodies

Antibodies used	SARS-CoV-2 N protein antibody clone 1C7C7 (Cell Signaling Technology, Cat #68344) was used at 1:300, and a AF594-conjugated Goat-anti-mouse secondary (ThermoFisher, Cat #A11005) at 1:100.
Validation	1. SARS-CoV-2 N antibody by supplier and by PMID: 32398876<br>2. AF594-conjugated Goat-anti-mouse secondary by supplier and by PMID: 35654839

Eukaryotic cell lines

Policy information about cell lines and Sex and Gender in Research

Cell line source(s)	We used the following cell lines: Vero E6-TMPRSS2-T2A-ACE2 (BEI, Cat# NR-54970); Vero E6 cells from ATCC (CRL-1586)
Authentication	Morphology for the cell line was assessed by microscope. Permissiveness of the cell line was assessed through observation of CPE by microscope and through quantification of virus produced by plaque assay.
Mycoplasma contamination	Cell lines were tested and negative for mycoplasma.
Commonly misidentified lines<br>(See ICLAC register)	None

Animals and other research organisms

Policy information about studies involving animals; ARRIVE guidelines recommended for reporting animal research, and Sex and Gender in Research

Laboratory animals	C57BL/6J and K18-hACE2 C57BL/6J (strain B6.Cg-Tg(K18-ACE2)2Prlmn/J) mice (Jackson Laboratories, https://www.jax.org/strain/034860) were used in this study. All animals were housed and bred in our AAALAC-accredited research facility. Rodent housing rooms are maintained at a temperature range of 21 - 23°C with a humidity range of 30-70% and a 12:12-h light:dark cycle. Housing room air exchange rates are set at 10 -15 air changes per hour. Mice are provided ad libitum autoclaved water and irradiated feed (5058 irradiated rodent chow, LabDiet, St. Louis, MO). Water is filtered prior to autoclaving. All mice are housed in autoclaved individually ventilated caging (Tecniplast, West Chester, PA) at 50-70 cage-volume air changes hourly. Cages are filled with 1/8 –in. corncob bedding (Bed-o-Cobs, Anderson, Maumee, OH) and each rodent cage receives nesting material (Nestlet, Ancare Corp., Bellmore, NY). Colony health surveillance is performed quarterly using a combination of dirty bedding sentinels and exhaust air dust testing. The following viral, bacterial, and parasitic pathogens are excluded from the rodent colony including mouse parvovirus (1-5), minute virus of mice, mouse hepatitis virus, mouse norovirus, Theiler's murine encephalomyelitis virus, epizootic diarrhea of mice, Sendai virus, pneumonia virus of mice, reovirus, Mycoplasma pulmonis, lymphocytic choriomeningitis virus, mouse adenovirus, ectromelia, K virus, polyomavirus, mouse cytomegalovirus, hantavirus, E. cuniculi, CAR Bacillus, mouse thymic virus, lactate dehydrogenase elevating virus, Clostridium piliforme, Helicobacter, fur mites and pinworms.<br><br>In order to produce neonatal heterozygous K18-hACE2 C57BL/6J mice for the transmission experiments, C57BL/6J females were bred with homozygous K18-hACE2 C57BL/6J males. Pups were housed with their mother for the duration of all experiments and infected at 4-7 days of age.<br><br>For adult mouse challenge (Supplemental Figure 1), we used 13-week-old heterozygous K18 mice.
Wild animals	No wild animals were used in this study.
Reporting on sex	For adult mouse challenge (Supplemental Figure 1), mice of both sexes were used, as indicated in the Source Data file. Sex of neonatal mice (4-7 days of age) could not be determined. Thus, presumably both sexes were used for all the infection and transmission studies.
Field-collected samples	No field-collected samples were used in this study.
Ethics oversight	Animal experiments were performed in the Animal Biosafety Level 3 (ABSL3) facility of NYU Grossman School of Medicine (New York, NY), in accordance with its Biosafety Manual and Standard Operating Procedures (IBC22-000088, Dittmann), and following Institutional Animal Care and Use Committee (IACUC) guidelines (IA18-00071, Dittmann).

Note that full information on the approval of the study protocol must also be provided in the manuscript.
